# Characterization of the GNSS RFI Threat to DFMC GBAS Signal Bands

**DOI:** 10.3390/s22228587

**Published:** 2022-11-08

**Authors:** Nadezda Sokolova, Aiden Morrison, Anja Diez

**Affiliations:** SINTEF Digital, Strindveien 4, 7034 Trondheim, Norway

**Keywords:** GNSS, RFI, GBAS, jamming, multi-frequency

## Abstract

This article presents analysis results from a long-term multi-site Global Navigation Satellite System (GNSS) Radio Frequency Interference (RFI) monitoring campaign in the context of Ground Based Augmentation System (GBAS) Dual Frequency Multi Constellation (DFMC) concept operation. GBAS resilience against unintentional RFI is an important area for investigation as the ground station receivers often must operate adjacent to high-traffic roads where chances of being affected by RFI are high. To be able to develop algorithms and reaction strategies necessary to ensure continuity and availability of service, knowledge of interference signal characteristics and frequency band/bands affected, as well as relative occurrence rates between the considered frequencies and frequency combinations, is necessary. The analysis presented in the article covers the prevalence and properties of the RFI events observed on the GPSs L1 and L5 and the Galileo E1 and E5a frequency bands that are considered by the on-going DFMC GBAS concept development initiatives. Due to being spectrally adjacent, the observed event analysis is also carried out for the Galileo E5b and GLONASS G1 frequency bands. The article also addresses the issue of spectral occupancy distribution of the observed events and presents new interesting RFI event types captured during the considered monitoring period.

## 1. Introduction

Although GNSS signals used in aviation are located in a protected Aeronautical Radio-Navigation Signal (ARNS) frequency band, unfortunately, jamming attacks affecting this band do occur, and with increasing rate [1,2,3]. Over the past several years, the combination of ubiquitous, low-cost communications systems and satellite navigation has moved civil Global Navigation Satellite System (GNSS) positioning and timing into use domains where there are stronger motivations for an attack. In particular, widespread use in road-tolling/automotive insurance or asset-tracking/fleet-management systems encourages attacks directed at GNSS. As GNSS becomes more deeply embedded into digital infrastructure, and low-cost GNSS receiver manufacturers already support dual- or multi-frequency solutions, we can expect to see more jamming events targeting multiple GNSS frequencies at the same time, as well as potentially more attacks of increased sophistication.

The Ground Based Augmentation System (GBAS) is a GNSS-based precision approach guidance system for the final approach phase. The system is intended to be used for safety-critical operations (e.g., zero visibility operations including Autoland), and is therefore designed to support very stringent integrity, continuity and availability requirements. Since GBAS receivers have to operate in close proximity to high-traffic roads and airport parking, the chances of being affected by Radio Frequency Interference (RFI) are high [4,5].

The evolution of GBAS Approach Service Types (GASTs) as well as development of new concepts/system architectures is a complex and resource intensive process since the development must be carried out in accordance with rigorous standards. At the moment, relevant standards for implementation are available only for GASTs C and D, Categories I and III, respectively, both based only on the use of the Global Positioning System (GPS) L1. At the same time, a number of research and industrial activities are focusing on developing new generation architectures and concepts based on the use of GPS L1 and L5 and Galileo E1 and E5a [6,7,8,9]. GBAS resilience against unintentional RFI is an important area for investigation. To be able to develop algorithms and reaction strategies necessary to ensure continuity and availability of service, as well as new system concepts relying on multiple frequencies (e.g., switching between modes based on different core frequencies), knowledge of jamming signal characteristics (center frequency, bandwidth, time modulation frequency of the signal, etc.) and frequency band/bands affected, as well as relative occurrence rates between the considered frequencies and frequency combinations, is highly beneficial.

RFI monitoring, including event classification and characterization, is an active area of research in the GNSS community, and there is a growing amount of material publicly available; see, for example, [1,10,11]. To study the RFI threat with regard to GBAS ground station operation and address the needs of both the GPS L1-based GAST D and the future dual-frequency, multi-constellation GBAS concepts, it was necessary to carry out a long-term multi-site monitoring campaign using equipment that would not only cover the frequency bands of interest but also allow for flexible analysis of the captured raw RFI event data (e.g., possibility to reprocess the captured events using different parameters of interest). To facilitate such monitoring and data analysis tasks, the low-cost multi-band advanced GNSS RFI detection, analysis and alerting system (ARFIDAAS) developed by SINTEF was used [12,13]. The system simultaneously supports all navigation bands transmitted by GPS (L1, L2 and L5), Galileo (E1, E5a, E5b and E6), GLONASS (G1, G2 and G3) and Beidou (B1, B1-2, B2 and B3). Detected RFI events are characterized, and the raw data are captured and sent for further analysis to the cloud storage. The centralized cloud storage allows for flexible and broad analysis of all captured events. To direct the analysis on frequency bands relevant for the GBAS GS operations, the captured RFI event data were processed and analyzed focusing on the GPSs L1 and L5, and the Galileo E1 and E5a frequency bands. Additionally, the observed event statistics were also studied for the Galileo E5b and GLONASS L1 frequency bands. This is mainly due to the fact that those frequencies are spectrally adjacent to and within the same ARNS bands as L5/E5a and L1/E1, respectively, so that a strong interference signal affecting Galileo E5b or GLONASS L1 can potentially have an impact on core signals depending on the filtering level carried out by the receiver.

This article describes the basic structure of the detection and classification steps carried out by the ARFIDAAS monitoring system and presents the results based on the RFI events captured at six different sites. Each of the selected sites was continuously monitoring the environment in terms of RFI in the L-band for over two calendar years.

## 2. RFI Data Capture and Event Classification

### 2.1. RFI Monitoring Network

**Figure 1:**
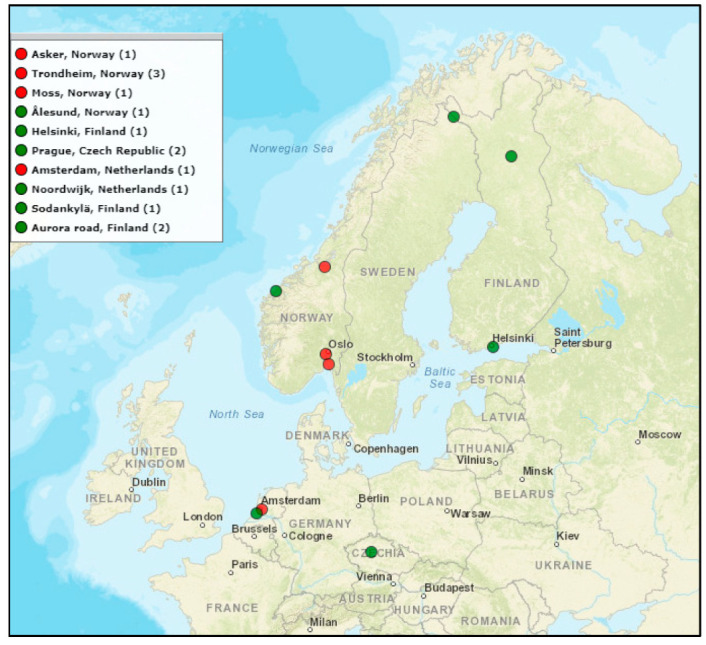
Locations of presently deployed RFI monitoring systems, with the number of sites indicated in brackets. Sites used in the analysis presented herein are shown in red.

At the moment of this article’s preparation, the RFI monitoring network included in total 14 stations; see Figure 1 showing the locations. As the station deployment was carried out gradually, the period of continuous monitoring at each location varies. For the analysis presented in this paper, a subset of six stations was selected based on the length of the operational period. Each of the stations is located in close proximity to heavy traffic roads, parking facilities and other transportation infrastructure. It is noted, however, that both the distance and the elevation angle between the monitoring station antenna and the road vary between sites. Typically, the antenna is installed in a roof-top location as it is shared with other GNSS research activities, resulting in a ‘below the horizon’ or negative elevation angle for RFI signals from vehicle-borne sources.

### 2.2. RFI Event Detection

First, RFI event detection is carried out based on deviation from a moving average power level rather than a static threshold of signal amplitude, which eliminates the need for calibration while also tolerating gain variation within the antenna and signal propagation network that can arise from temperature variation. Second, the center frequencies, Intermediate Frequency (IF) bandwidths and sampling rate parameters can each be adjusted to tailor the amount of the captured spectrum to the signal bands and bandwidths supported by the connected antenna. Third, the system software allows for some masking of nuisance events either in terms of reporting, uploading, or both in cases where sites are found to suffer from the presence of a persistent yet unstable in-power co-authorized user that would otherwise cause frequent nuisance detections. Fourth, the spectral shape of the ‘environment baseline’ is noted, and deviations from this are used to determine which region of the band is affected. Examples of sources that can cause such nuisance detections include Radio Detection And Ranging (RADAR) installations, amateur radio, amateur television, malfunctioning Wi-Fi routers, etc. It is noted that, for the purpose of this study, the absolute power level of the detected RFI was not controlled, and all events which exceed the site-specific dynamic triggering thresholds were considered for the purpose of determining RFI populations and characteristics. For a more detailed description of system design, the reader is referred to [12,13,14]. If during event analysis no substantial change in band shape or apparent power allocation within the band is detected, the event is classified as an environment baseline event. Such events can be caused by wideband noise sources which cover the monitored band uniformly but with a limited amount of power, such that peaks from the main lobes of GNSS signals, particularly in the L1 band where these are quite visible, remain.

### 2.3. Automatic Event Classification

**Figure 2:**
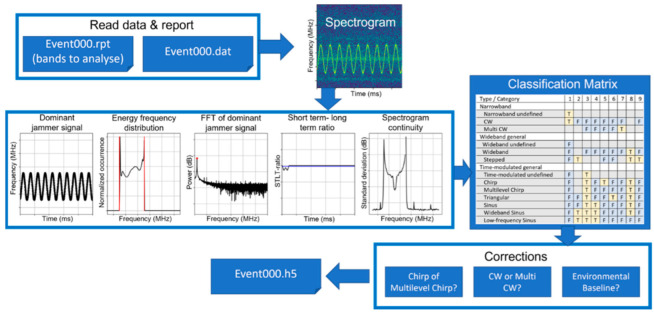
Automated RFI event characterization, classification and processing (adapted from [15]).

In support of efficient system operation, an algorithm for automatic event classification was developed [15]. The algorithm performance was first validated using simulated jamming events with known characteristics, and then using the real data collected by the monitoring stations shown in Figure 1. Figure 2 illustrates the basic flow of the classification process. This process is now continuously running at each of the monitoring stations where the classification results, including the identified jamming signal type for each detected event, are saved together with parameters such as the center frequency, bandwidth and time modulation frequency of the jammer signals and reported monthly to the site-holder. The accumulated classification results are then used for long-term statistical analysis. In this article, only a brief description of the process is provided; for additional implementation details, as well as discussion of the challenges encountered, see [15,16].

**Figure 3:**
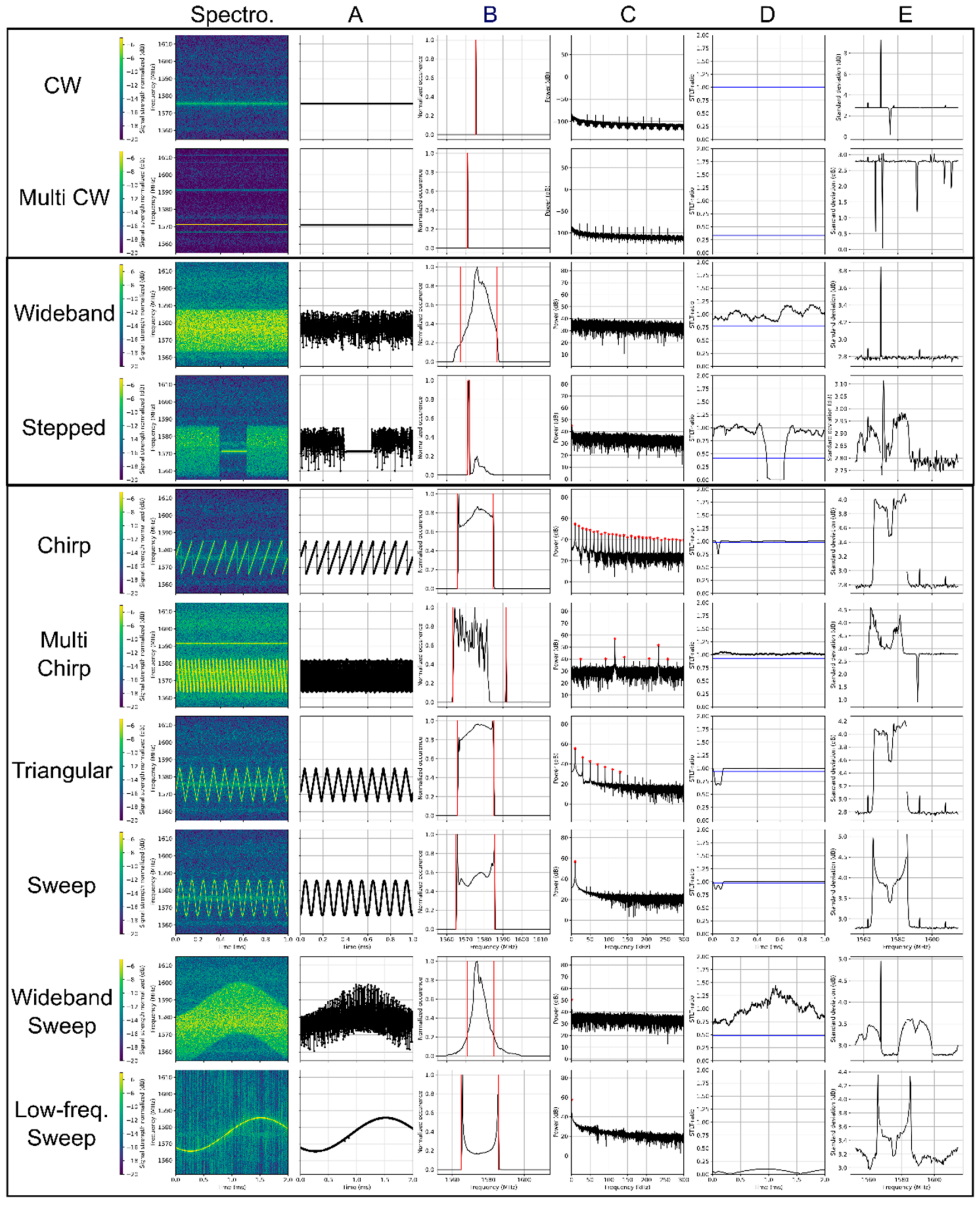
Overview over the identified RFI subcategories, their appearance in the spectrogram and the derived functions (**A**–**E**) for distinguishing the different classes (adapted from [15]).

Five main functions were derived from the spectrogram to classify the RFI events and derive their bandwidth, center frequency and repetition frequency in the case of time-modulated signals:

- Dominant jamming signal (Figure 3A);
- Energy frequency distribution (Figure 3B);
- Fast Fourier Transform (FFT) of jamming signal (Figure 3C);
- Short-term–long-term (STLT) ratio (Figure 3D);
- Spectrogram continuity (Figure 3E).

For classification of the detected RFI events, three main categories were defined: narrowband signals, wideband signals and time-modulated signals with multiple subcategories for each as shown in Figure 3. To be more specific, the narrowband signals are defined to have a bandwidth narrower than 0.5 MHz over the observation window. The majority of these signals are continuous wave (CW)-type, but undefined narrowband signals which include an element of time modulation in spectrum or power are also observed. In the case of events with multiple CW signals at different frequencies, the challenge is to detect all these separate CW signals, especially if they have a significant difference in relative power level. While only the strongest CW event might be visible in the dominant jamming signal (Figure 3A), continuous signals and their frequency can be identified in the spectrum continuity function (Figure 3E). Additionally, as the standard deviation of the energy increases for all jamming signal types, except for CW events, this function is also utilized by the algorithm to identify multiple CW events, even in the presence of other jammer signal types.

Wideband signals are defined as signals with a bandwidth larger than 0.5 MHz, without a measurable time-varying frequency content. The classification algorithm is capable to distinguish wideband signals and stepped signals. Identifying stepped signals is somewhat challenging due to the large variety of signal types that can be defined as a stepped signal. Here, the focus is on signals that are narrowband signals of short time intervals detectable in the short term long term ratio (STLT) function (Figure 3D). Stepped signals with a regular reoccurrence might also have a time-modulated frequency that will be detected in the FFT of the dominant jammer signal (Figure 3C); they are nevertheless categorized as wideband signals.

Time-modulated signals are in general also wideband but have a regularly recurring time-modulated component, such as for example a sinus function. This recurrence of the signal is detectable in the FFT of the dominant jammer signal as one or more resonance peaks (Figure 3C). Sinus signals, whether general, wideband, or low-frequency, have exactly one resonance peak. In the case of chirp signals and triangular signals, multiple resonances (fundamental and harmonics) with regular spacing and reduction in energy with increasing frequency can be observed. The spacing of the resonances (harmonics) is a multiple of the fundamental frequency for chirp signals and a multiple of twice the fundamental frequency for triangular signals. In the case of a multilevel chirp jammer, the jammer event does not sweep linearly through a frequency range. This is visible in the FFT of the jammer signal as side resonances with lower energy compared with the fundamental resonance and its harmonics (Figure 3C, multi chirp).

Making use of the differences in the defined five functions described above for the different jamming signal types, nine evaluation criteria are defined and are used to distinguish between the different jamming signal classes.

## 3. Data Analysis and Results Discussion

Information about each detected event’s RFI modulation, bandwidth, center frequency, power level and sweep rate where applicable is saved along with the raw IF sample data. The results discussed below are all formed using an aggregation of the observed parameters from each site. As mentioned above, the statistical analysis of the accumulated RFI event data presented herein is based on data from six of the stations within the overall network, specifically the three sites located in Trondheim (Norway), the Asker site (Norway), the Amsterdam site (Netherlands) and the Moss site, also in Norway. These sites were selected as they have been in continuous operation for one or more years. In total, about 8.5 full years of data were used (in particular, 3116 days), during which 18,600 RFI events were detected. It is noted that this total number of observed events includes the observations of the L1/E1 and L5/E5a/E5b frequencies only. Thus, the number is higher if other frequency bands (L2, E6, etc.) are to be included. Readers interested in the analysis presented herein but focusing on the Galileo E6 frequency band are referred to the material provided in [14,17].

Aviation GNSS receivers have to follow strict interference rejection requirements that are defined in the form of interference rejection masks for each frequency band covering both the wideband and continuous wave (CW) interference signal types [18,19]. Since ARFIDAAS stations are hosted at locations with various RF network configurations, they do not report absolute power levels but instead rely on detecting variations from the local baseline. For this reason, while enough data are captured to determine absolute power levels at the receiving antenna, the rejection requirement masks are not considered in this study, though are an ongoing activity.

### 3.1. L1/E1, L5/E5a and E5b Frequency Bands

Table 1 shows the frequency band limits considered in the analysis focusing on the L1/E1, L5/E5a and E5b bands. The values were selected to cover the frequency band areas with potentially no suppression applied at the antenna level [20,21].

**Figure 4:**
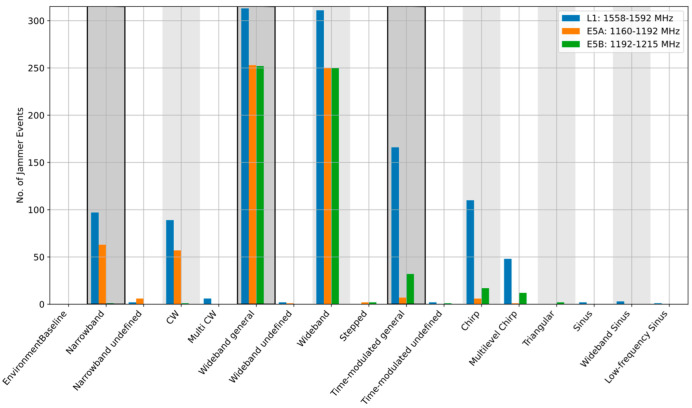
Detailed classification results, Asker station, Norway, 2020.

**Figure 5:**
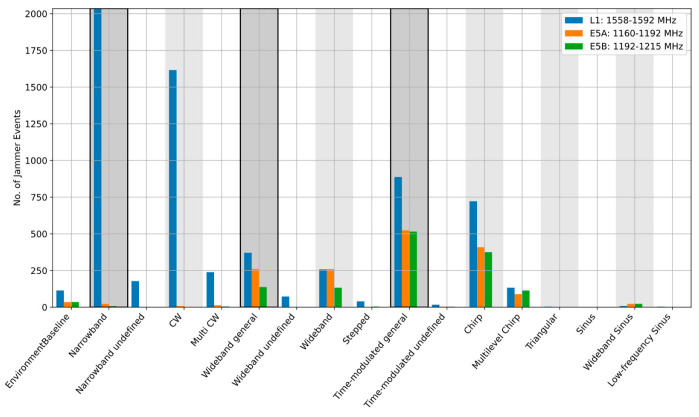
Detailed classification results, Moss station, Norway, 2021.

**Figure 6:**
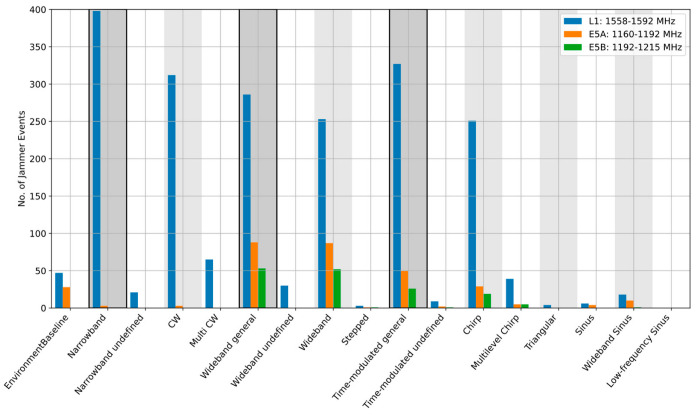
Detailed classification results, Amsterdam station, Netherlands, 2020.

When the event parameters are aggregated over longer observation periods, site-specific RFI environment features become visible. It is also possible to see which jamming signal type is prevalent for the frequency band of interest. As Figure 4, Figure 5 and Figure 6 illustrate, the dominant type of RFI varies from site to site. In 2020, the Asker site, for example, was dominated by the wideband noise on the L1/E1 band, whereas the Amsterdam and Moss sites were clearly dominated by the narrowband events, such as continuous wave (CW) and multi-CW sources on L1/E1. An interesting observation is that the situation on other bands differs. Table 2 provides a summary of the dominant interference types at each side for each frequency band. It is noted that in Table 2, the dominant RFI type is defined as the category with the largest number of observed events. An additional RFI type is also indicated in the case when one or more other categories are equal to or above 70% of the dominant one.

Table 3 shows the results summarized per site considering the L1/E1, E5a and E5b frequency bands. On average, RFI was present for 23.4 s per day with most of the events affecting the L1/E1 band (21.7 s per day). The second mostly affected frequency appears to be the E5a with 5.5 s per day closely, followed by the E5b frequency band affected for 4.8 s per day on average. Based on the accumulated data, it appears that in the majority of cases, if there is interference on the L5/E5a frequency band, the L1/E1 band is also impacted. The situation is similar in the case with the L1/E1 and E5b combination, however, the number of events impacting the E5b band alone is slightly higher.

As stated earlier, and can as well be observed from this table, there is a substantial site-to-site variation, not only in terms of the dominant interference type per site and band as shown in Table 2, but also in the accumulated number of seconds with RFI present and ratios between frequency bands. While at some sites (e.g., Trondheim) RFI on L1/E1 is clearly prevalent, on others (e.g., Asker), the ratio between L1/E1 and L1/E1 vs. E5a bands is 2.5 to 1.

Another interesting observation gathered from the long-term dataset analysis is that the relative likelihood of different GNSS carriers being impacted at a given station shows extreme variability, even at the level of full-month observation periods, suggesting that for accurate site characterization a minimum measurement campaign of several months may be necessary. Our assumption is that this is potentially due to individual site statistics being heavily influenced by a small number of jamming devices which tend to revisit the site, which is conceptually consistent with vehicle-borne personal privacy devices (PPDs) operated by individuals with daily routines, or the temporary presence of fixed emission sources in range of the monitoring station.

Table 4 summarizes the observed ratios between the considered frequency bands alone and in combinations. The ratio values are calculated based on the observed number of seconds per day per site per band/band combination averaged across all sites used in this analysis. Probability of occurrence results are summarized in Table 5, where the values are also calculated based on the time (i.e., number of seconds) RFI was observed for per site and on average. It is noted that no site-dependent weighting was applied for average value calculation.

**Figure 7:**
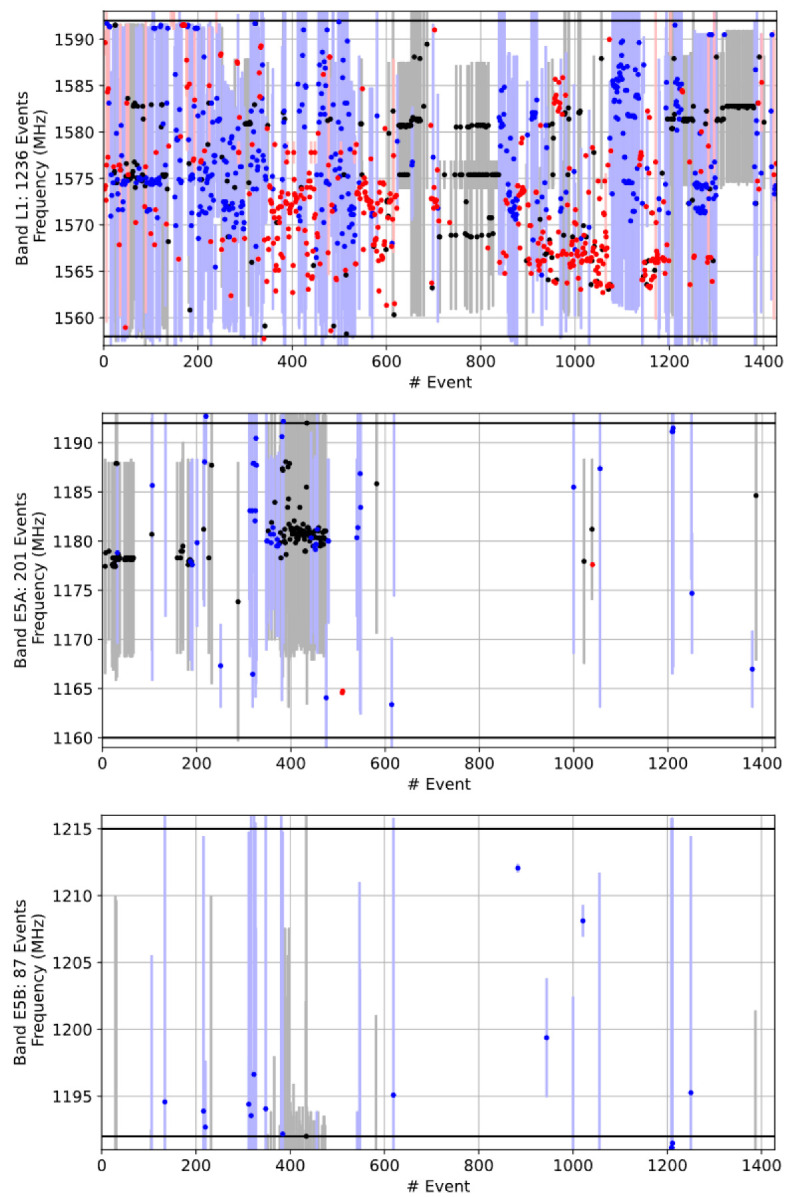
Band occupancy, Amsterdam station, Netherlands, 2020. Events that occur at the same time at different bands are given the same event number. The center frequency of each event is marked with a dot and the bandwidth with a vertical line. Narrowband events are indicated with red, wideband black and time-modulated blue. Black horizontal lines indicate the band limits.

**Figure 8:**
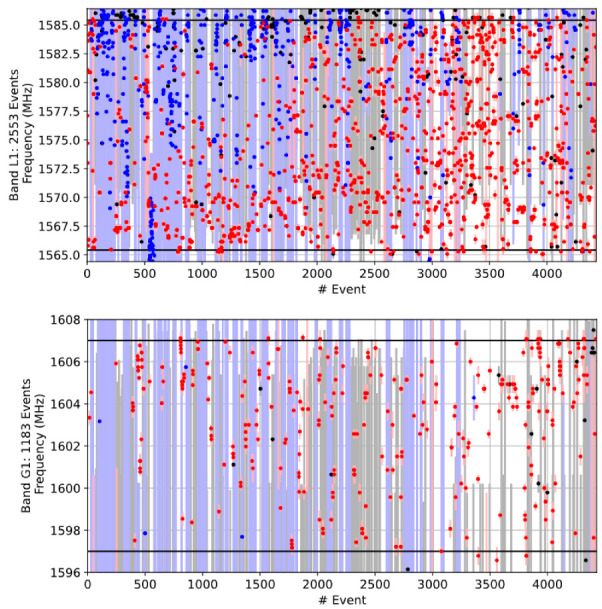
Band occupancy, Moss station, Norway, January-March 2022. Events that occur at the same time at different bands are given the same event number. The center frequency of each event is marked with a dot and the bandwidth with a vertical line. Narrowband events are indicated with red, wideband black and time-modulated blue. Black horizontal lines indicate the band limits.

Another interesting observation based on the long-term monitoring results analysis is that for four of the six sites considered, the category of narrowband noise on the E1/L1 bands is either the dominant type or is within 70% of the dominant one. The current working theory for this disparity is that the vast majority of these narrowband detections are emissions from low-cost active E1/L1 GNSS receiver devices which have become self-resonant or otherwise leak energy in this band. This assumption was confirmed in several cases when the interference source was localized and the signal source identified by the spectrum management authorities in Norway (see Section 4.3 in reference [3] discussing specific field cases). When considering the distribution of narrowband sources versus spectrum location in Figure 7, where the narrowband events are visualized in red, and the categorization in Figure 6, we can see that the Amsterdam station observes vastly more narrowband interference in the E1/L1 band than the other bands considered. It is noted that vertical lines in Figure 7 and Figure 8 indicate the bandwidth of each of the observed RFI events. In the case of narrowband interference, the vertical lines represent the extent of a multi CW event type that is considered as narrowband but is covering a region of the spectrum.

If this phenomenon was unrelated to the specific E1/L1 frequency band and was instead uniformly distributed over the L-band, then we would expect to see approximately two times more events over the other monitored bands than on E1/L1, but this is obviously not the case. While it is conceptually uncomfortable to consider that GNSS receivers may be a large source of GNSS RFI, this might also imply that as low-cost multi-frequency GNSS receivers proliferate that we should expect the prevalence of narrowband RFI in each of the other bands to increase over time.

### 3.2. L1/E1 and G1 Frequency Bands

In addition to the analysis presented in the section above, an analysis attempting to map the RFI event observations made on GPS/Galileo L1/E1 vs. GLONASS G1 frequency bands was also carried out. For this particular case, band limits as shown in Table 6 were used. This choice was made in order to study what types of RFI and how often comparatively events will be observed on these two bands considering the same amount of bandwidth used (±10 MHz). The data and monitoring site subset used in this case were the same as detailed above.

First, the dominant interference signal types were studied, (summarized results shown in Table 7), where it was observed that the amount of the narrowband interference on G1 is far lower than that on L1/E1, with about only every fourth narrowband event impacting the G1 frequency. This is consistent with the belief that the narrowband RFI is primarily generated by malfunctioning GNSS receivers. It is entirely reasonable that a large number of legacy GPS-only L1 receivers/systems may still be in use on the Scandinavian/European roadways. The same observation can also be made from the L1/E1 and G1 band occupancy plots shown in Figure 8 considering the Moss monitoring station for a period of 3 months in early 2022.

We also studied the interference presence per day at each of the monitoring sites on these two bands. The average ratio between the amount of RFI on L1/E1 and G1 is 2.2. Very few events affecting both bands simultaneously were detected. Another interesting observation is that if comparing the results for the L1/E1 band with narrower band limits of ± 10 MHz to ± 17 MHz used earlier (see Table 5), the amount of RFI decreases with a factor of 1.5. Based on high-level analysis, the outcome that fewer events occur within the narrowed monitoring range, affecting all RFI families (i.e., narrowband, time-modulated and wideband), is entirely expected.

### 3.3. New RFI Type Observations

**Figure 9:**
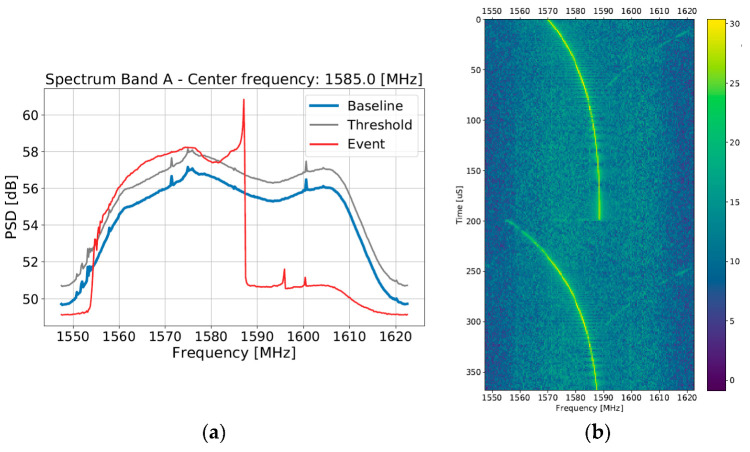

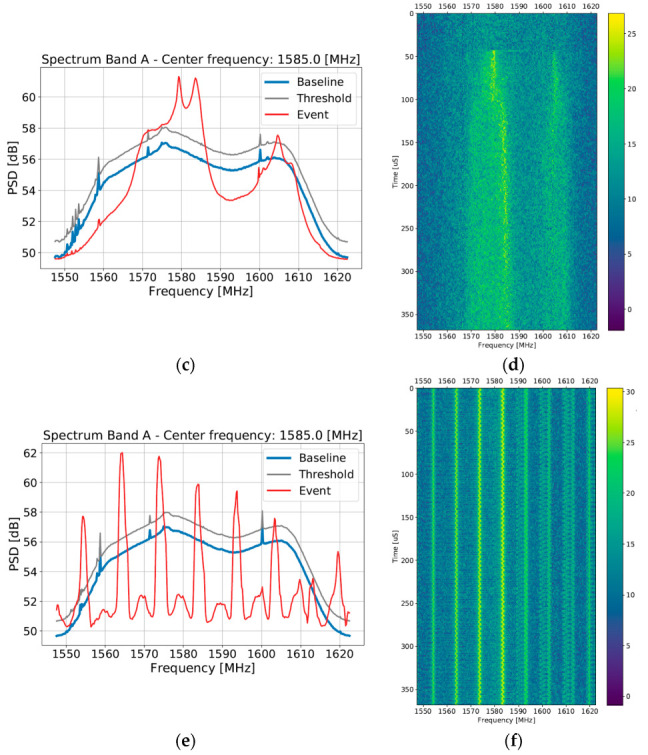
Examples of (**a**,**b**) wideband time-modulated signal using an exponential function; (**c**,**d**) non-stationary distribution of RFI potentially evidencing meaconing or spoofing; (**e**,**f**) modulated rake/multi-CW.

GNSS RFI jamming signal characteristics have been studied extensively by many researchers [22,23,24]. Throughout the observation period used for this analysis, a number of RFI signal types which were not previously observed in the wild were captured. These include, but are not limited to, signals that were simultaneously low bandwidth, yet chirp-modulation in fine-grained structure, potentially originating from poorly designed jamming devices (or self-oscillator equipment with a chirp-like instability), and those that were wideband time-modulated signals that appear to use an exponential sweeping rather than a linear function as their sweep modulation signal (Figure 9a,b).

Figure 9c,d shows the onset of an RFI event which shows a transient onset of wideband RFI with two lobes concentrated around the L1 and G1 center frequencies, which is consistent with a potential meaconing or spoofing event. Figure 9e,f shows an example of a rarely seen modulated rake signal where each of the spurs follows the same frequency toggling behavior in an apparently stable pattern.

**Figure 10:**
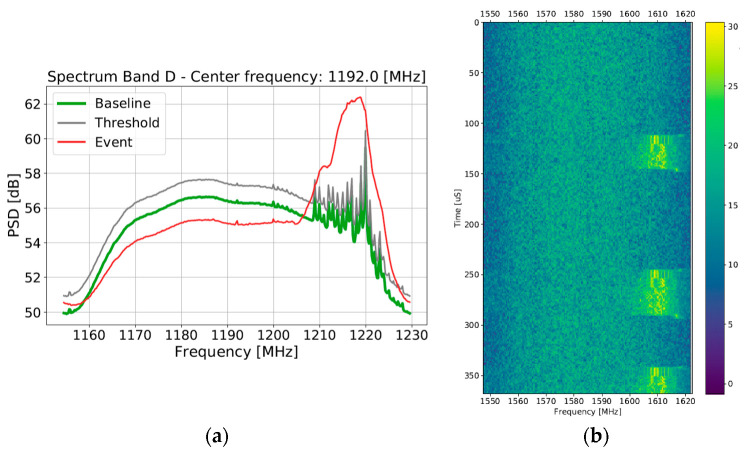
Examples of (**a**,**b**) a malfunctioning Wi-Fi router radiating at half of the nominal frequency of operation.

Figure 10a,b is considered separately as, unlike the events shown in Figure 9 which all impact the L1/E1 band, the event in Figure 10 instead intrudes upon the E5b signal. The signal in this case shows transitions between multiple modulation modes at the scale of tens of microseconds in discrete bursts. Each burst appears to start and end with a short period of narrowband activity, followed by an orthogonal frequency division multiplexing (OFDM) or narrowband rake RFI with several distinct subcarriers visible, then wideband modulation over the impacted band. Based on help from the spectrum management authorities in Norway, this source was identified as originating from a malfunctioning Wi-Fi router with a failure mode causing it to radiate at half the intended frequency. Disturbingly, lab evaluation of such devices shows that the radio emitted more power in the E5b band than in the 2.4 GHz band, with only the antenna tuning limiting the L-band emissions.

## 4. Conclusions and Recommendations

The analysis presented in this article covered RFI event characterization, RFI threat profile update and statistical analysis of the captured RFI events in terms of probability of occurrence, occurrence rate on the L1/E1 frequency only and L5/E5a frequency only, as well as L1/E1 and L5/E5a frequency bands together. Results obtained on the adjacent bands such as the Galileo E5b and GLONASS L1 were also analyzed and presented for consideration.

As the basis for the analysis, data captured at multiple RFI monitoring stations in Scandinavia/Europe were used, totaling 8.5 site-years of aggregate observation (i.e., periods for all stations used in the study summed together). It is believed that the site diversity and the number of observations used in the exercises (18,600 events) provide sufficient and representative statistics to confidently illustrate the current RFI threat. It is noted, however, that as none of the sites used in the analysis are located directly at the airport premises, the observed ratios between bands and the observed trend in the increasing number of unintentionally generated events, as well as the new observed signal types, are to serve as indications of the RFI threat space evolution required to support initial decisions regarding potential reaction and mitigation strategies. The following conclusions were drawn based on the analysis results presented above:

- Both the number of events observed, as well as the amount of time per day with interference present, are the highest on the L1/E1 frequency band on all sites used in the study.
- The L1/E1 frequency band is impacted most by the assumingly unintentional narrowband interference caused by malfunctioning GNSS equipment. As low-cost multi-frequency navigation receivers proliferate, it is believed that the total rate of the narrowband EMI in the L5/E5a and E5b bands will increase as a consequence, potentially reaching parity with the rates now observed in L1/E1.
- RFI events affecting L5/E5a only are less common. In the majority of cases, when there is interference on L5/E5a, L1/E1 is also affected (1.3 to 1 E5a to L1/E1 + E5a ratio).
- The average ratio of RFI on L1/E1 vs. RFI on L5/E5a is approximately 3.9 to 1. The ratio for RFI on L1/E1 vs. RFI on both L1/E1 and L5/E5a is approximately 5.1 to 1.
- Both the total number of events and dominant type of the interference signal, as well as the impact ratios (L1 vs. L5, L1 vs. L1, L5, etc.), depend strongly on the site and time period considered.
- When the combination of wideband and time-modulated sources is considered, they become the majority of events detected at the majority of the sites (here, all sites except for Moss, Norway). In all cases, the combination is within the same order of magnitude as the narrowband events, and therefore should not be ignored.
- Significant month-to-month variation was observed in both total RFI occurrence and the impact ratios.
- GNSS signal jamming is a dynamic/evolving threat. While several of the new jamming signal types observed within this monitoring campaign are variations of existing modulations and concepts, e.g., exponentially swept vs. linearly swept, this should be taken into account when/if considering use of in-receiver mitigation schemes.

Based on the observations discussed above, we arrive at several high-level recommendations:

- The prevalence of RFI is higher than initially expected and could be a factor for continuity. Based on the observation that the GBAS system has low tolerance to loss of C/N0 before observations are discarded by low-signal-power monitor, and lower starting C/N0 due to characteristics of MLAs, antennas siting relative to roadways are believed to be very important in light of the RFI occurrence rate results.
- Given the observed average ratio of 1 to 3.9 of the interference on L5/E5a vs. interference on L1/E1, inclusion of an additional fallback mode should be subjected to a cost–benefit analysis given that the proportion of events which affect the L5/E5a band only is within an order of magnitude of the occurrence rate on L1/E1.
- While a histogram of in-band power levels was not presented here, this should be evaluated when considering ground antenna siting, as distance alone may be insufficient to meet system Electro-Magnetic Interference (EMI) assumptions. Alternately, it may be necessary to rely on earthworks or other obstructions to local roadways and other transportation infrastructure.
- While new types of jamming signals observed within this monitoring period are variations of existing modulations and concepts, e.g., exponentially swept vs. linearly swept, this should be taken into account when/if considering use of in-receiver mitigation schemes.

One important factor to keep in mind is that the class of jamming/interference attacks considered, namely the low-cost in-car jamming devices, will potentially change in the future where the ratio of interference on L1/E1 and L5/E5a might change. The trend in the number of RFI events simultaneously affecting both bands can change as well following the developments in the low-cost multi-frequency GNSS receiver marked. An additional detail to note is that most of the environment monitoring was performed during the 2020–2021 period, during which the RFI environment was affected by what appear to be changes in individual behavior and road traffic brought on by the corona virus lockdowns and restrictions on mobility. A higher probability of occurrence is therefore expected in the future.

## Figures and Tables

**Table 1:** L1/E1, L5/E5a and E5b frequency band limits used in the analysis.

Frequency Band	Band Limits (MHz)
L1/E1	1558–1591
L5/E5a	1160–1192
E5b	1192–1215

**Table 2:** Summary of the dominant RFI type per site for the L1/E1, E5a and E5b frequency bands.

Site	L1/E1	L5 (E5a)	E5b
Moss	Narrowband	Time-modulated	Time-modulated
Trondheim	Narrowband/Time-modulated	Time-modulated	Time-modulated
Trondheim B	Time-modulated	Wideband	Wideband
Trondheim C	Time-modulated/Narrowband	Wideband/Time-modulated	Wideband/Time-modulated
Asker	Wideband	Wideband	Wideband
Amsterdam	Time-modulated/Narrowband/Wideband	Wideband	Time-modulated

**Table 3:** Site activity summary considering the L1/E1, E5a and E5b frequency bands.

			Average RFI Presence per Day (Seconds)						
Site	Days of Observation	Number of Events	All Bands Accumulated	L1/E1	L5/E5a	E5b	L1/E1 + E5a	L1/E1 + E5b	L1/E1 + E5a + E5b
Moss	463	5670	30.2	29.3	7.2	5.5	6.0	4.9	4.6
Trondheim	561	2551	17.3	15.6	2.1	3.0	1.4	1.4	1.3
Trondheim B	535	909	7.8	7.5	1.5	1.7	1.3	1.4	1.3
Trondheim C	730	5016	37.9	32.4	11.2	10.7	6.8	5.7	5.6
Asker	342	1444	19.2	17.9	7.3	6.6	6.0	6.4	5.7
Amsterdam	485	2183	27.7	27.6	3.9	1.5	3.8	1.5	1.4
Total	3116	18,600							
Average			23.4	21.7	5.5	4.8	4.2	3.6	3.3

**Table 4:** Observed RFI occurrence ratios between L1/E1, E5a and E5b frequency bands alone and combined.

Occurrence Ratios					
L1/E1 vs. L5/E5a	L1/E1 vs. E5b	L1/E1 vs. L1/E1 + L5/E5a	L1/E1 vs. L1/E1 + E5b	L1/E1 vs. L1/E1 + L5/E5a + E5b	E5a vs. E5b
3.9	4.5	5.1	6.1	6.5	1.1

**Table 5:** Probability of RFI occurrence per site and band/band combination considering the L1/E1, E5a and E5b.

	Probability of RFI Occurrence					
Site	L1/E1	L5/E5a	E5b	L1/E1 + L5/E5a	L1/E1 + E5b	L1/E1 + E5a + E5b
Moss	3.34 × 10^−4^	8.30 × 10^−5^	6.37 × 10^−5^	7.00 × 10^−5^	5.65 × 10^−5^	5.32 × 10^−5^
Trondheim	1.80× 10^−4^	2.42 × 10^−5^	3.52 × 10^−5^	1.5 × 10^−5^	1.57 × 10^−5^	1.47 × 10^−5^
Trondheim B	8.67 × 10^−5^	1.74 × 10^−5^	1.96 × 10^−5^	1.53 × 10^−5^	1.66 × 10^−5^	1.53 × 10^−5^
Trondheim C	3.75 × 10^−4^	1.30 × 10^−4^	1.24 × 10^−4^	7.91 × 10^−5^	6.63 × 10^−5^	6.54 × 10^−5^
Asker	2.07 × 10^−4^	8.40 × 10^−5^	7.58 × 10^−5^	6.96 × 10^−5^	7.46 × 10^−5^	6.55 × 10^−5^
Amsterdam	3.20 × 10^−4^	4.52 × 10^−5^	1.76 × 10^−5^	4.44 × 10^−5^	1.72 × 10^−5^	1.68 × 10^−5^
Unweighted average	2.50 × 10^−4^	6.39 × 10^−5^	5.60 × 10^−5^	4.90 × 10^−5^	4.12 × 10^−5^	3.85 × 10^−5^

**Table 6:** Frequency band limits used to analyze event occurrence on GPS/Galileo L1/E1 vs. GLONASS G1.

Frequency Band	Band Limits (MHz)
L1/E1	1565–1585
G1	1592–1612

**Table 7:** Summary of the dominant RFI type per site for the L1/E1 and G1 frequency bands.

Site	Dominant RFI Type on L1/E1 (1575.42 MHz ± 10 MHz)	Dominant RFI Type on G1 (1602.00 MHz ± 10 MHz)
Moss	Narrowband	Time-modulated/Narrowband
Trondheim	Narrowband/Time-modulated	Time-modulated
Trondheim B	Time-modulated/Narrowband	Time-modulated
Trondheim C	Time-modulated/Narrowband	Time-modulated
Asker	Wideband	Wideband

## Data Availability

The ARFIDAAS RFI library is available to interested research and corporate parties free of charge beyond the provision of storage media and/or payment for download bandwidth from the cloud storage solution. To obtain data access please contact one of the authors.
